# Working memory updating in individuals with bipolar and unipolar depression: fMRI study

**DOI:** 10.1038/s41398-022-02211-6

**Published:** 2022-10-11

**Authors:** Anna Manelis, Yaroslav O. Halchenko, Lisa Bonar, Richelle S. Stiffler, Skye Satz, Rachel Miceli, Cecile D. Ladouceur, Genna Bebko, Satish Iyengar, Holly A. Swartz, Mary L. Phillips

**Affiliations:** 1 Department of Psychiatry, Western Psychiatric Hospital, University of Pittsburgh Medical Center, University of Pittsburgh, Pittsburgh, PA USA; 2 Department of Psychological and Brain Sciences, Dartmouth College, NH USA; 3 Department of Statistics, University of Pittsburgh, Pittsburgh, PA USA

**Keywords:** Learning and memory, Bipolar disorder, Depression, Human behaviour

## Abstract

Understanding neurobiological characteristics of cognitive dysfunction in distinct psychiatric disorders remains challenging. In this secondary data analysis, we examined neurobiological differences in brain response during working memory updating among individuals with bipolar disorder (BD), those with unipolar depression (UD), and healthy controls (HC). Individuals between 18–45 years of age with BD (*n* = 100), UD (*n* = 109), and HC (*n* = 172) were scanned using fMRI while performing 0-back (easy) and 2-back (difficult) tasks with letters as the stimuli and happy, fearful, or neutral faces as distractors. The 2(*n*-back) × 3(groups) × 3(distractors) ANCOVA examined reaction time (RT), accuracy, and brain activation during the task. HC showed more accurate and faster responses than individuals with BD and UD. Difficulty-related activation in the prefrontal, posterior parietal, paracingulate cortices, striatal, lateral occipital, precuneus, and thalamic regions differed among groups. Individuals with BD showed significantly lower difficulty-related activation differences in the left lateral occipital and the right paracingulate cortices than those with UD. In individuals with BD, greater difficulty-related worsening in accuracy was associated with smaller activity changes in the right precuneus, while greater difficulty-related slowing in RT was associated with smaller activity changes in the prefrontal, frontal opercular, paracingulate, posterior parietal, and lateral occipital cortices. Measures of current depression and mania did not correlate with the difficulty-related brain activation differences in either group. Our findings suggest that the alterations in the working memory circuitry may be a trait characteristic of reduced working memory capacity in mood disorders. Aberrant patterns of activation in the left lateral occipital and paracingulate cortices may be specific to BD.

## Introduction

Highly disabling bipolar and major depressive (also called unipolar depression [UD]) disorders affect 60 million Americans and cost the United States more than $150 billion annually [1, 2]. Individuals suffering from these disorders spend half of their lives with depression and/or mania [3–6]. Depression is characterized by sad mood, anergia, sleep disturbances, and feelings of guilt and worthlessness. Mania is characterized by elated or irritable mood, excessive energy/activity, and impulsivity. These symptoms impair cognitive and emotional functioning and affect job performance, school work, and social relationships [3–6]. Understanding the distinct neurobiological characteristics of cognitive dysfunction in related psychiatric disorders remains challenging because multiple psychiatric disorders share common patterns of disruption in brain regions supporting cognitive functioning [7].

Working memory is involved in maintenance and real-time manipulation (i.e., updating) of information in a person’s mind [8]. It is critical for schooling, work, driving, and even social relationships. Difficult working memory tasks usually require more cognitive resources than easy tasks, so people perform these tasks more slowly and less accurately than easy tasks [9, 10]. Working memory updating can be measured using n-back tasks in which participants are required to match the stimuli they see on the screen with those they saw one, two, or three trials previously. Poorer working memory updating is related to poorer problem-solving ability [11], and elevated rumination characterizing depressive disorders [12]. Although disruption of working memory is a common cognitive deficit in Bipolar Disorder (BD) and UD [13–17], studies often report inconsistent findings and rarely include both diagnostic groups. For example, some studies reported that manic but not depressed individuals with BD had significant deficits in working memory [18], that euthymic individuals with BD did not differ from Healthy Controls (HC) [19], and that individuals with UD did not differ from HC [20]. However, other studies demonstrated that both individuals with BD and UD, compared to HC, showed slower and/or less accurate performance on working memory tasks [21, 22] with UD outperforming BD [22].

Difficult working memory tasks are associated with greater activation in prefrontal cortical (PFC), anterior cingulate, and posterior parietal regions than easy working memory tasks [23, 24]. Aberrant functioning of the PFC, anterior cingulate, and parietal cortices may underlie the working memory deficits in mood disorders [25–30] and even help distinguish BD from UD [31, 32]; however, controversies about specific alterations persist even within a single diagnostic category. Some fMRI studies report that compared to HC, individuals with mood disorders show greater activation in the PFC when the working memory task becomes more difficult [29, 30, 33], while other studies report opposite findings [34]. For example, individuals with BD, compared to HC, had smaller difficulty-related changes in the PFC activation [35]. Consistent with these findings, a recent meta-analysis examining neural substates of the n-back task reported reduced activation in lateral and medial PFC in individuals with BD compared to HC, but greater activation in the right posterior parietal cortex and superior frontal cortex in individuals with UD vs. HC [36]. However, other meta-analyses were unsuccessful in identifying reliable differences between individuals with mood disorders and HC. For example, one meta-analysis comparing brain activation patterns characterizing individuals with UD vs. HC found no significant differences between these two groups [37]. Likewise, a meta-analysis that examined the differences between euthymic individuals with BD vs. HC found no significant differences in brain activation during n-back tasks after family-wise error (FWE) correction [19].

The inconsistencies in the behavioral and neuroimaging findings reported by meta-analyses and individual studies of mood disorders could be explained by high heterogeneity in participant samples, small sample sizes of individuals studies (often less than 25 participants per group), and methodological differences across studies (e.g., different scanning sequences, cognitive tasks, and analysis pipelines). In this secondary analysis, we aimed to identify neurobiological differences between individuals with BD, UD, and HC using one of the largest datasets ever collected in a single laboratory using the same working memory task [25, 38], similar eligibility criteria, and similar clinical assessments across the studies. Working memory was examined using the n-back task with letters as the stimuli and happy, fearful, and neutral faces as distractors [25, 38]. The distractors were introduced to examine the participants’ ability to inhibit irrelevant emotionally salient (happy and fearful faces) and emotionally ambiguous (neutral faces) information. Previous research suggested that individuals with BD and UD differ in their response to emotional faces [39, 40] with the former showing reduced performance when processing positive emotional stimuli and the latter showing reduced performance when processing negative emotional stimuli [40]. The differences in emotion processing could differentially affect the ability of individuals with BD and UD to resolve interference from emotional stimuli especially during a difficult working memory task. Considering these differences as well as the evidence for more severe cognitive deficit in BD than UD [22, 41] and smaller difficulty-related activation changes in working memory regions in BD versus HC [35], we hypothesized that the PFC, anterior cingulate, and parietal brain regions would show smaller difficulty-related activation changes in individuals with BD versus UD and HC, especially in the presence of happy faces. However, based on the meta-analytical findings [37], we did not expect to find differences between individuals with UD and HC.

## Method

### Participants

In this study, we performed secondary data analysis that combined n-back behavioral and neuroimaging data collected in three previous studies in the same laboratory (Table 1). All studies were approved by the University of Pittsburgh Human Resource Protection Office. Participants were recruited from community, universities, counseling, and medical centers through referrals and advertisements between 2009 and 2018. All participants signed informed consent to participate in the study. Table 1 describes the studies, participants, and the neuroimaging data acquisition parameters.

**Table 1:** Description of the studies included to the analyses.

Study/Funding/PI	Study description	Age	N
BPA2 R01MH076971 PI: Phillips	*Toward the Identification of Biomarkers of Bipolar Disorder*. This completed project examined biological markers of BD type-I that reflect pathophysiologic brain processes common to depression and remission and specific to bipolar disorder. *Dx*: BD, UD, HC *Scanner*: 3T Siemens Trio at the MRRC at the University of Pittsburgh *Acquisition parameters*: MPRAGE: voxel = 1 mm^3^, TR = 2200 ms, 192 slices. EPI: voxel = 3.2 mm^3^, TR = 2000 ms, 39 slices	18–45	168
COBY R01MH059929 MPIs: Birmaher, Phillips, Versace	*Course and Outcome of Bipolar Disorder in Youth*. This ongoing project aims to determine how previous clinical course of BD and treatment exposure from childhood into adulthood impacts neural circuitry functioning and structure in individuals with BD. *Dx*: BD, HC *Scanner*: 3 T Siemens PRISMA at the MRRC at the University of Pittsburgh *Acquisition parameters*: MPRAGE: voxel = 1 mm^3^, TR = 1520 ms, 176 slices. EPI: MB = 3, voxel = 2.3 mm^3^, TR = 1500 ms, 54 slices	18–35	54
DIAMOND R01MH100041 PI: Phillips	*Reward, Pathophysiologic Dimensions and Psychological Distress in Young Adults*. This ongoing project aims to identify relationships among functioning in working memory and other neural circuitry and various dimensions of psychopathology in young individuals seeking help for psychological distress. *Dx*: BD, UD, HC *Scanner*: 3 T Siemens Trio or PRISMA at the MRRC at the University of Pittsburgh *Acquisition parameters*: MPRAGE: voxel = 1 mm^3^, TR = 2200 ms, 192 slices. EPI on PRISMA: MB = 3, voxel = 2.3 mm^3^, TR = 1500 ms, 54 slices. EPI on TRIO: voxel = 3.2 mm^3^, TR = 2000 ms, 39 slices	18–25	253

All participants were fluent in English and the majority were right-handed. HC had no personal or family history of psychiatric disorders. Symptomatic individuals met DSM-IV or DSM-5 criteria for either BD or UD. To be included into the studies, participants had to be within the age range appropriate for the study (Table 1) and be free of neurological, endocrine, and other systemic illnesses at the time of scan. Exclusion criteria applied to all participants included history of severe head trauma, systemic medical illness that could impact fMRI measures of cerebral blood flow, standard exclusion criteria for MRI scanning (e.g., claustrophobia, surgically implanted ferromagnetic devices and objects in/on the body, weight > 300 lbs), current pregnancy, premorbid IQ < 85 per the National Adult Reading Test (NART) [42], substance use disorder in the past 6 months (lifetime for HC) or current use of illicit substances as determined by the Structured Clinical Interview (SCID) and pre-scan saliva alcohol and urine drug screens, inability to understand or speak English, visual disturbance (<20/40 acuity in the Snellen test), and borderline personality disorder per SCID-II. Individuals with BD were excluded if they met criteria for a current manic/hypomanic episode.

The original samples included 168 participants from the BPA2 study, 253 participants from the DIAMOND study, and 54 participants from the COBY study. The quality assurance (image quality, excessive motion during scan, poor n-back task performance, less than 2 usable runs, or any combination of the above reasons) was passed by 136 participants from the BPA2 study (BD = 62, HC = 35, UD = 39), 38 participants from the COBY study (BD = 33, HC = 5), and 207 participants from the DIAMOND study (BD = 5, HC = 132, UD = 70). Thus, a total of 381 participants (BD = 100, UD = 109, HC = 172) were included in the data analyses. Table 2 reports demographic and clinical characteristics of the samples.

**Table 2:** Demographic and clinical characteristics.

	BD	UD	HC	statistics
N	100	109	172	
Gender (number females)	66 (66%)	81 (74.3%)	110 (64%)	χ^2^ (2) = 3.4, *p* = 0.2
Study:				
• BPA2	62	39	35	χ^2^(4) = 166.03, *p* < 0.001
• COBY	33	0	5	
• Diamond	5	70	132	
Scanner:				
• Trio	63	72	111	χ^2^(2) = 0.21, *p* = 0.8
• Prisma	37	37	61	
Age (years)	29.33 (0.76)	24.85 (0.63)	24.38 (0.45)	*F*(2,378) = 19.51, *p* < 0.001 BD > UD: t(207) = 4.57, *p* < 0.001 BD > HC: t(270) = 5.97, *p* < 0.001 UD = HC: t(279) = −0.6, *p* = 0.54
IQ (NART)	110 (1.18)	110.4 (0.77)	110 (0.53)	*F*(2,368) = 0.08, *p* = 0.9 BD = UD: t(202) = −0.3, *p* = 0.8 BD = HC: t(260) = 0, *p* = 0.99 UD = HC: t(274) = −0.5, *p* = 0.66
Education:				
• College or above	30	37	67	chi = 6.02(4), *p* = 0.2
• Some college	50	53	87	
• High school or less	20	19	19	
Handedness (left hand)	5	0	0	
Illness Onset	15.39 (0.73)	17.07 (0.44)	na	BD < UD: t(207) = −2.01, *p* = 0.046
Illness Duration	13.95 (0.66)	7.77 (0.61)	na	BD > UD: t(207) = 6.84, *p* < 0.001
Current depression severity (HAMD-17)	11.13 (0.82)	18.01 (0.61)	2.23 (0.28)	*F*(2,377) = 246.45, *p* < 0.001 BD < UD: t(206) = −6.84, *p* < 0.001 BD > HC: t(269) = 12.32, *p* < 0.001 UD > HC: t(279) = 26.41, *p* < 0.001
Current mania severity (YMRS)	3.51 (0.33)	3.15 (0.21)	0.54 (0.09)	*F*(2,377) = 76.07, *p* < 0.001 BD = UD: t(206) = 0.9, *p* = 0.3 BD > HC: t(269) = 10.63, *p* < 0.001 UD > HC: t(279) = 13.01, *p* < 0.001
State anxiety (STAIY1)	41.24 (1.39)	51.33 (1.01)	30.32 (0.71)	*F*(2,376) = 124.7, *p* < 0.001 BD < UD: t(205) = −5.95, *p* < 0.001 BD > HC: t(268) = 7.76, *p* < 0.001 UD > HC: t(279) = 17.47, *p* < 0.001
Trait anxiety (STAIY2)	44.91 (1.39)	56.54 (0.92)	32.27 (0.77)	*F*(2,369) = 168.62, *p* < 0.001 BD < UD: t(200) = −7.16, *p* < 0.001 BD > HC: t(261) = 8.63, *p* < 0.001 UD > HC: t(277) = 20.02, *p* < 0.001
Number of participants taking psychotropics	66 (66%)	33 (30.3%)	1 (0.6%)	
A mean total medication load	2.21 (0.22)	0.87 (0.16)	0.01 (0.01)	*F*(2,378) = 74.91, *p* < 0.001 BD > UD: t(207) = 5, *p* < 0.001

For continuous measures entries are mean and standard error of mean (SE).

*ns* not significant, *na* not applicable.

### Clinical assessments

All participants were administered the SCID for DSM-IV or DSM-5 disorders [43, 44]; the Hamilton Depression Rating Scale (HAMD-17) [45] to assess the severity of depression during the last 1 week; Young Mania Rating Scale (YMRS) [46] to assess the severity of mania during the last 1 week; and State and Trait Anxiety Inventory (STAIY1, STAIY2) [47] to assess the severity of state and trait anxiety.

### Behavioral assessment

#### Experimental paradigm

**Fig. 1:**
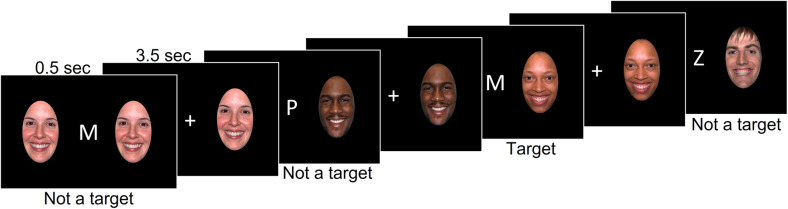
Emotional faces n-back task. The figure illustrates the 2-back condition with happy face distractors.

All participants performed the emotional faces n-back task [25, 38] in which they were presented with a sequence of letters. In the 0-back condition, participants had to press the response button when the letter ‘M’ appeared on the screen. In the 2-back condition, they had to press the response button when they saw a stimulus that was the same as the stimulus presented two trials ago (Fig. 1). Each block of n-back was presented either without distractors, or with happy, fearful, or neutral face distractors taken from the NimStim dataset [48]. The distractors were presented on the right and left sides of the letter. Participants were asked to ignore the faces because they were irrelevant to the n-back task. Participants completed 3 runs of the task in the BPA2 study and 2 runs in the COBY and DIAMOND studies. Each run consisted of eight blocks of trials (0-/2-back × no face/happy/fearful/neutral faces). Each block was preceded by a 4000-ms instruction screen informing about either 0-back or 2-back task conditions. A block included twelve 500-msec trials that were separated by jittered inter-trial intervals whose mean duration was 3500 msec. Participants were instructed to respond as quickly and accurately as possible whenever a target stimulus appeared on the screen.

#### Behavioral data analysis

Participants’ RT and accuracy were averaged per participant, *n*-back and emotional condition and evaluated using Group (BD/UD/HC) x n-back (0-back/2-back) × emotion (happy/neutral/fearful) mixed effects models (R packages *‘lme4’* [49], *‘lmerTest’* [50], and *‘psycho’* [51]) with age, IQ, and sex as covariates and participant as a random effect. The means and contrasts between conditions of interest were estimated from the mixed effect models using the ‘*modelbased’* package [52] in R. When appropriate, the *p*-values were corrected for multiple comparisons using Benjamin and Hochberg’s False Discovery Rate (FDR) [53].

### Neuroimaging

#### Data acquisition

The acquisition details are described in Table 1.

#### Preprocessing

The Digital Imaging and Communications in Medicine (DICOM) images were converted to the Brain Imaging Data Structure (BIDS) with *heudiconv* [54] using ReproIn heuristic [55]. The neuroimaging data quality was examined using *mriqc* 0.15.1 [56]. The data were preprocessed using *fmriprep* 20.1.1 [57]. Preprocessing steps included skull-stripping of T1w images, brain surface reconstruction using recon-all (FreeSurfer 6.0.1) [58], and brain masks generation. For each BOLD image, we applied motion correction, spatiotemporal filtering using *mcflirt* [59], and slice-timing correction using 3dTshift [60]. Preprocessing also included automatic removal of motional artifacts using ICA-AROMA [61], spatial smoothing with an isotropic, Gaussian kernel of 6 mm FWHM (full-width half-maximum), the BOLD image registration to the MNI template, and regressing out non-steady state volumes. High-pass temporal filter with 100 s cutoff was applied on the *fmriprep* preprocessed files.

#### 1st-level and 2nd-level analyses

In the 1^st^-level analysis, explanatory variables included the no face distractor, and happy, fearful, and neutral face distractor conditions in 0-back and 2-back tasks, the instruction screens, and the motor response to account for the fact that only a fraction of trials (30%) required a motor response. The contrasts for 2-back minus 0-back were calculated for each emotional condition to determine the differences in brain activation for difficult vs. easy working memory tasks. A hemodynamic response was modeled using a Gamma function. The mean difference in brain activation between 2-back and 0-back tasks for available runs was calculated for each participant/emotional condition during the 2nd-level analysis.

#### Group-level analysis

The 2-back minus 0-back contrasts computed for each participant and each emotional condition during the 2nd-level analysis were used as inputs to the Sandwich Estimator (*swe*) [62], the approach used for nonparametric permutation inference for longitudinal and repeated measures neuroimaging data. The *swe* estimated a 3 (groups: HC/BD/UD) by 3 (emotions: happy/fear/neutral) model with scanner, study, age, sex, and IQ as covariates. Considering that the group analysis combined participants from different studies that were scanned on 2 different scanners, we orthogonalized the design matrix using the package ‘*matlib*’ in R (https://github.com/friendly/matlib) with a QR decomposition by Gram-Schmidt orthonormalization. The matrix included the variables in the following order: scanner, study, age, sex, IQ, and the columns modeling diagnostic groups and emotional conditions. The n-back conditions without face distractors were modeled at the 1st-level analyses but were not included into the group analysis because the effect of specific emotional distractor on the group differences could be hindered by a strong effect of a face presence (vs. no face). Although the main effect of emotions was included into the model for completeness, it was outside the focus of this paper and, therefore, the results are reported in Supplemental Materials. The *swe* was conducted in the whole brain with Threshold-Free Cluster Enhancement correction (TFCE) [63] and 5000 permutations. The outcome variables were the three F-test maps (a main effect of group, a main effect of emotion and a group-by-emotion interaction). The FWE p-value threshold was set to *p* < 0.01 to account for the three F-tests using the Bonferroni correction (0.05/3 = 0.0167).

All further analyses were conducted in R (https://www.r-project.org) using percent signal changes extracted using *featquery* for each participant and task condition from the brain regions identified in the group analysis described above. The FDR correction for multiple tests [53] was applied when appropriate. We compared the 2-back-minus-0-back differences for BD vs. UD, BD vs. HC, and UD vs. HC groups using mixed effect models with the percent signal changes as a dependent variable, and Group as an explanatory variable. Cohen’s d or partial eta2 were calculated as appropriate. Also, we used the mixed effect models to predict the 2-back-minus-0-back differences in RT and accuracy (separately) from the interaction between BD/UD/HC diagnostic status and the 2-back-minus-0-back differences in brain activation in the regions identified in the *swe* analysis. In all models, age, IQ, and sex were covariates and participants were a random effect.

### Exploratory analyses

The goal of exploratory analyses was to investigate whether the differences in clinical measures between BD and UD differentially affect behavioral and brain activation outcomes in these individuals. We examined the interaction effect between diagnostic status (BD/UD) and current depression (HAMD-17), mania (YMRS), and anxiety (STAIY) symptoms as well as illness duration, mood state, and a total psychotropic medication load on the 2-back-minus-0-back differences in RT, accuracy, and brain activation in individuals with mood disorders (BD/UD). Age, IQ, and sex were covariates and participants were a random effect. Missing values in the HAMD-17 and YMRS assessments (one value per assessment) were imputed using the ‘*mice’* package [64] in R.

## Results

### Clinical

A total of 381 participants passed f/MRI and behavioral data quality assurance. The missing IQ values for 10 participants were imputed as the sample mean. The results of the BD vs. UD vs. HC demographic and clinical characteristics comparisons are reported in Table 2. Compared to individuals with UD, individuals with BD were older, had earlier illness onset, longer illness duration, lower current depression scores based on HAMD-17 but higher current mania score based on YMRS. They also had higher state and trait anxiety, and higher total medication load. Based on the HAMD-17, 40% of those with BD were euthymic (score < 8), 36% mildly depressed (score 8–16), 14% moderately depressed (score 17–23), and 10% severely depressed (score > 24). was Among those with UD, 5% were euthymic (score < 8), 30% mildly depressed (score 8–16), 46% moderately depressed (score 17–23), and 19% severely depressed (score > 24).

### Behavioral

Supplemental Figure S1 illustrates RT for correct responses and accuracy. There was a group-by-*n*-back interaction (*F*(2,1895) = 10.3, *p* < 0.001), as well as the main effects of n-back (*F*(1, 1895) = 195.8, *p* < 0.001) and group (*F*(2, 376) = 11.3, *p* < 0.001) on accuracy. More accurate responses were observed in younger participants (*F*(1,376) = 6.8, *t* = −2.6, *p* = 0.01) and those with higher IQ (*F*(1,376) = 18.8, *t* = 4.3, *p* < 0.001). There was no significant relationship of accuracy with sex (*p* = 0.83). Contrasts estimation with the FDR correction showed that HC were significantly more accurate than individuals with UD during 0-back (*t* = 2.4, p-FDR-corrected = 0.023), and more accurate than those with BD (*t* = 5.9, *p*-FDR-corrected < 0.001) and UD (*t* = 3.76, p-FDR-corrected < 0.001) during 2-back. Individuals with UD were more accurate than those with BD during 2-back (*t* = 2.16, p-FDR-corrected = 0.038). There was no significant main effect of distractor face emotions or interactions between emotion and other variables on accuracy.

There was a group-by-*n*-back interaction (*F*(2,1895) = 11.9, *p* < 0.001), as well as the main effects of *n*-back (*F*(1, 1895) = 864.9, *p* < 0.001) and group (*F*(2, 376) = 4.8, *p* = 0.008) on RT. Faster responses were observed in younger participants (*F*(1,376) = 21.0, *t* = 4.6, *p* < 0.001). There was no significant relationship of RT with sex (*p* = 0.97) or IQ (*p* = 0.06). Contrast estimation showed that the three groups did not differ from each other in their RT on the 0-back task. On the 2-back task, HC were significantly faster than individuals with BD (*t* = −3.9, p-FDR-corrected < 0.001) and UD (*t* = −3.5, p-FDR-corrected < 0.001) who did not differ from each other. There was no significant main effect of distractor face emotions or interactions between emotion and other variables on RT.

### Neuroimaging

**Fig. 2:**
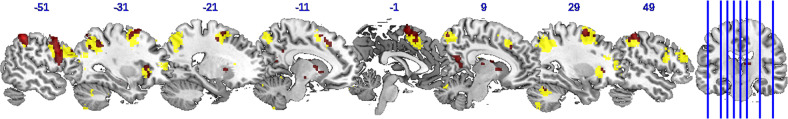
The F-test results for the main effect of group (in red) thresholded at *p* < 0.01. It is overlaid over the working memory circuitry derived from the NeuroSynth meta-analysis posterior probability map (in yellow) thresholded at *p* < 0.001.

**Fig. 3:**
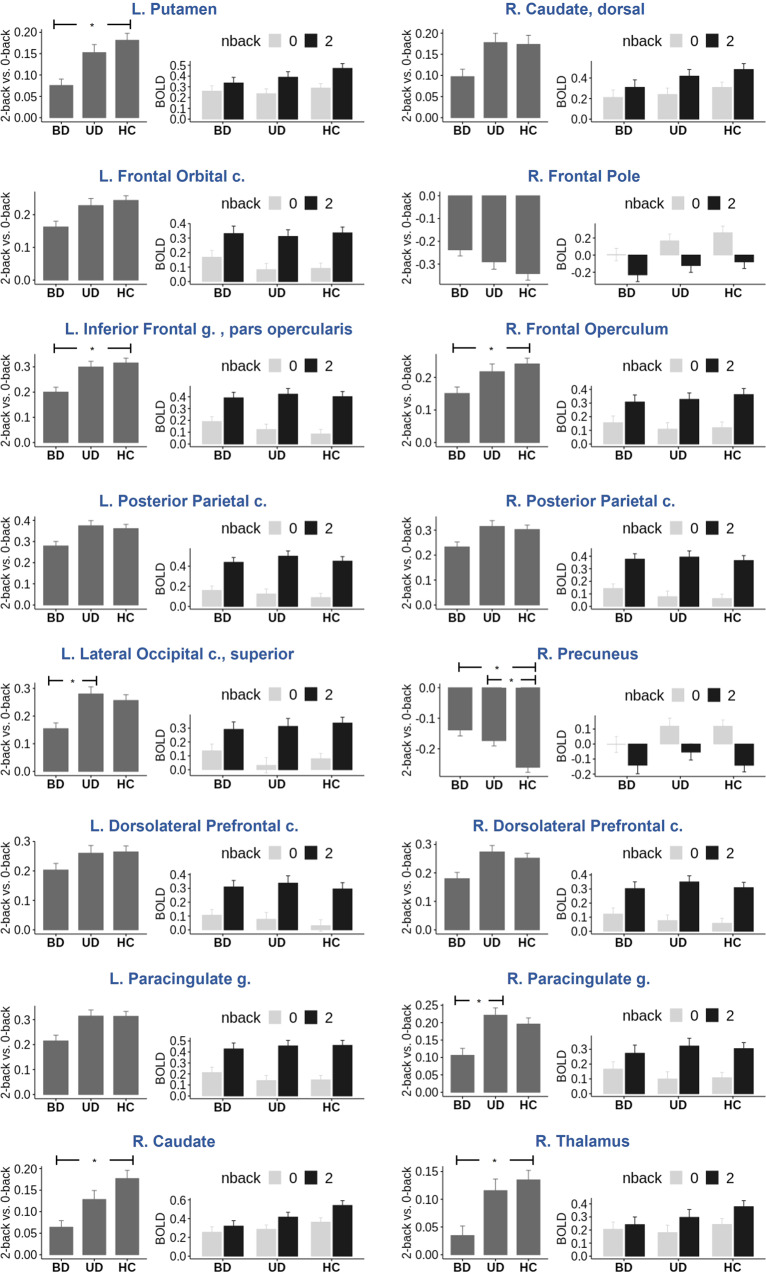
Percent signal changes in each region of interest (ROI) showing the main effect of group. For each ROI, the plot on the left shows the 2-back vs. 0-back differences in activation, while the plot on the right shows percent signal changes separately for 0-back and 2-back.

There were main effects of group (Figs. 2, 3, Table 3) and emotion (Supplemental Fig. S2, Supplemental Table S1) but no group-by-emotion interaction effect on the 2-back minus 0-back differences in brain activation. The regions identified in the whole brain analyses almost completely overlapped with those in the working memory circuitry derived by the NeuroSynth meta-analysis [65] from 1091 studies (Fig. 2), thus suggesting that the regions we identified pertain to the working memory circuitry.

**Table 3:** Main effect of Group on 2-back vs. 0-back differences in brain activation.

R/L	Region	Number of voxels	Z-score in the peak voxel	The peak voxel coordinates in MNI space		
				*X*	*Y*	*Z*
L	Inferior Frontal gyrus, pars opercularis (LIFG)	1193	43.4	−46	10	26
L	Posterior Parietal cortex	919	56.2	−48	−46	52
R	Posterior Parietal cortex	501	36.3	40	−44	42
L	Paracingulate gyrus	432	38.6	−6	16	48
R	Frontal Operculum cortex	210	34.4	40	22	0
L	Putamen	206	36.2	−16	10	2
L	Frontal Orbital cortex	165	28.6	−34	26	−4
R	Dorsolateral prefrontal cortex (Middle/Superior Frontal gyrus) [DLPFC]	157	34.1	30	0	62
R	Precuneus	144	29.8	8	−58	20
L	Lateral Occipital cortex, superior division	70	25.7	16	−72	60
L	Dorsolateral prefrontal cortex (Middle Frontal gyrus) [DLPFC]	68	28.5	−46	30	32
R	Caudate	56	24.1	10	4	4
R	Paracingulate cortex	38	24	6	26	40
R	Caudate, dorsal	36	24.8	14	−2	18
R	Frontal Pole	31	29.1	2	66	12
R	Thalamus	23	20.6	8	−18	14

The analysis of pairwise comparisons of BD vs. UD, BD vs. HC, and UD vs. HC showed that although the nominal differences at uncorrected *p* < 0.05 between BD and UD were observed in most of the regions, only the left lateral occipital cortex, superior division and the right paracingulate cortex survived the FDR correction with BD showing significantly less pronounced 2-back vs. 0-back differences than UD. Individuals with UD were not different from HC in any of the identified regions except for the right precuneus that deactivated for 2-back vs. 0-back and in which HC had greater deactivation than individuals with UD. Individuals with BD had smaller 2-back vs. 0-back activation differences than HC in the left putamen and inferior frontal gyrus, pars opercularis (LIFG), and the right caudate, frontal opercular cortex, precuneus, and thalamus. The effect sizes were small even for the comparisons with highly significant FDR-corrected *p*-values (Supplemental Table S2).

Participants’ age was negatively associated with the 2-back vs. 0-back activation differences in the LIFG (F(1,373) = 11.7, *p* < 0.001), left lateral occipital cortex (*F*(1,373) = 14.4, *p* < 0.001), left paracingulate cortex (*F*(1,373) = 12.5, *p* < 0.001), and bilateral posterior parietal cortices (left: *F*(1,373) = 18.7, *p* < 0.001, right: *F*(1,373) = 17.6, *p* < 0.001). Younger participants had greater activation differences between 2-back and 0-back. There was no significant group-by-age interaction effect nor significant effects of sex, IQ, group-by-sex, or group-by-IQ interaction effects on the 2-back vs. 0-back differences in any of the 16 brain regions (Supplemental Table S3).

The 2-back minus 0-back differences in accuracy were explained by the interaction effect between Group and the 2-back minus 0-back activation differences in the right precuneus. This effect was driven by a significant negative association between the 2-back minus 0-back differences in brain activation and accuracy in the BD group (*t* = −3.5, *p* < 0.001). No such associations were observed in the UD (*t* = 0.9, *p* = 0.36) and HC (*t* = 0.6, *p* = 0.56) groups (Supplemental Table S4, Fig. S3).

The 2-back minus 0-back differences in RT were explained by the interaction effect between Group and the 2-back minus 0-back activation differences in the left lateral occipital cortex, left paracingulate cortex, right DLPFC, frontal opercular cortex, and right posterior parietal cortex. The effects were driven by a significant negative association between the 2-back vs. 0-back differences in brain activation and the 2-back vs. 0-back differences in RT in the BD group (left lateral occipital cortex: *t* = −3.5, *p* < 0.001; left paracingulate cortex: *t* = −4.0, *p* < 0.001; right DLPFC: *t* = −4.9, *p* < 0.001; frontal opercular cortex: *t* = −4.1, *p* < 0.001; right posterior parietal cortex: *t* = −2.7, *p* = 0.003). No such associations were observed in the UD (left lateral occipital cortex: *t* = 0.7, *p* = 0.5; left paracingulate cortex: *t* = 0.04, *p* = 0.97; right DLPFC: *t* = 0.2, p = 0.8; frontal opercular cortex: *t* = 0.3, *p* = 0.74; right posterior parietal cortex: *t* = 1.0, *p* = 0.3) and HC (left lateral occipital cortex: *t* = −1.3, *p* = 0.2; left paracingulate cortex: *t* = −0.02, *p* = 0.98; right DLPFC: *t* = −1.0, *p* = 0.3; frontal opercular cortex: −0.3, *p* = 0.76; right posterior parietal cortex: *t* = −0.9, *p* = 0.9) groups (Supplemental Table S5, Fig. S4).

### Exploratory analyses

The exploratory analysis conducted across the BD and UD groups revealed no main effects of illness duration, mood state, HAMD-17, YMRS, state/trait anxiety, or total medication load as well as no interaction between these clinical variables and Group (BD/UD) on the 2-back minus 0-back differences in RT, accuracy, or activation differences in either brain region (Supplemental Tables S6, S7).

## Discussion

In this study, we performed a secondary data analysis to examine working memory updating in a large sample of individuals with BD, UD, and HC across the three studies. Working memory updating was measured as the difference in behavioral (RT and accuracy) and brain responses in the 2-back (a difficult working memory task) compared to 0-back (an easy attentional task) tasks. Consistent with previous reports [66], HC were faster and more accurate than the individuals with BD or UD on the more difficult 2-back task, supporting a general deficit in working memory updating in mood disorders.

The three groups also differed from each other in the magnitude of brain activation changes between 2-back and 0-back in the bilateral PFC, posterior parietal, paracingulate, striatal regions as well as the left lateral occipital and the right precuneus and thalamic regions. Individuals with BD, compared to HC, showed smaller activation differences between 2-back and 0-back tasks in the bilateral striatum, LIFG, and the right frontal opercular cortex, thalamus and right precuneus. Individuals with BD, compared to those with UD, showed smaller activation differences in the left lateral occipital cortex and right paracingulate cortex. Individuals with UD, compared to HC, showed smaller activation differences in the right precuneus. Notably, greater reduction in accuracy for 2-back compared to 0-back was associated with smaller differences between 2-back and 0-back in the right precuneus, while greater worsening of RT for 2-back compared to 0-back was associated with smaller difference between 2-back and 0-back in the left lateral occipital cortex, left paracingulate cortex, right DLPFC, frontal opercular cortex, and right posterior parietal cortex in individuals with BD but not in those with UD or HC. Although the regions described above were identified in the whole brain analysis, they almost completely overlapped with the working memory circuitry determined by the NeuroSynth meta-analysis [65], thus suggesting that BD and UD affect functioning of the working memory circuitry critical for executive function and, specifically, working memory updating [8].

The findings of a negative association between the changes in behavioral performance and the changes in brain activation for 2-back, compared to 0-back, in BD suggest that worsening of accuracy and RT for a difficult working memory task might be related to inability to increase activation in the brain regions critical for working memory updating. In general, this idea is consistent with the previously proposed hypothesis that individuals with mood disorders, especially those with BD, have reduced working memory capacity compared to HC [35]. It is also consistent with the recently proposed model suggesting that patients with mood disorders may reach their maximum mental capacity at a lower load of cognitive tasks than HC [67].

Few studies compare neural correlates of working memory updating in individuals with BD, UD, and HC in the same study. Considering that these studies used different versions of the n-back task and had much smaller sample sizes, it is not surprising that our results were inconsistent with previous findings. For example, one study reported that individuals with UD had lower activation in the PFC compared to those with BD during the 1-back task [68]. Another study revealed that individuals with BD showed less deactivation in the medial frontal cortex than those with UD during the n-back task. Still another study suggested that the two groups of patients could be distinguished based on the activation differences for 2-back vs. 1-back tasks in the left DLPFC [32]. When comparing BD with HC, the results of our study were consistent with findings showing reduced difficulty-related activation changes in the PFC and parietal regions in individuals with BD compared to HC during the n-back task [26, 35, 69, 70], but inconsistent with the studies that either reported the opposite pattern of brain activation in BD [71] or no significant differences between these two groups [19]. Although a recent meta-analysis found no differences between individuals with UD and HC [37], our study, by contrast, found reduced difficulty-related difference in the right precuneus that showed greater deactivation in HC than UD. Our finding that both individuals with BD and UD deactivated right precuneus less than HC but did not differ from each other during 2-back vs. 0-back was inconsistent with the previous report of lower deactivation in BD than in UD in medial frontal cortex [72].

Our exploratory analyses aimed to investigate whether the differences in clinical characteristics between individuals with BD and those with UD could explain the differences between these groups of individuals in behavioral and brain correlates of working memory. Consistent with some previous reports [72], we found no main effect of participants’ mood state, current depression and mania symptoms, state and trait anxiety, or psychotropic medications load. In addition, we found no interaction effects between these clinical characteristics and Group on the 2-back-minus-0-back differences in accuracy, RT, or brain activation. These results were inconsistent with previous studies showing that DLPFC inversely correlated with HAMD-17 scores in individuals with BD [26]. Although older individuals had smaller differences in LIFG, left lateral occipital cortex, left paracingulate cortex, and bilateral posterior parietal cortices activation for 2-back vs. 0-back, these effects did not depend on diagnostic status. This result was inconsistent with previous reports suggesting that participants’ age may contribute to depression-related working memory impairments [21]. Taken together, our findings support a previously proposed idea that the alterations in the working memory circuitry may be a trait characteristic of mood disorders [69].

It was proposed that individuals with depression have aberrant cognitive processing because of their inability to focus on the task and, at the same time, direct attention away from negative thoughts they experience [73]. Based on this, we expected that different emotional distractors would affect behavioral and brain response in the n-back task more in individuals with mood disorders than in HC. Inconsistent with these predictions, the effect of distractors was not sensitive to diagnostic status. Although the bilateral MFG, and the left juxtaposition and superior parietal lobules showed the main effect of emotions, there was no significant interaction effect between diagnostic status and emotion of the face distractor on either RT, accuracy, or brain activation. One explanation for the lack of the interaction effect is that the n-back task fully engaged participants’ attention, so the effect of distractors was diminished. The other explanation is that although faces are processed automatically [74], the effect of mood disorder diagnosis can only be observed when deep processing of emotional faces, including recognition of emotional expressions, is required, which was not the case in this study.

Although group differences were observed in multiple regions across the working memory circuitry, it appears that the difficulty-related changes in the left lateral occipital and right paracingulate (sometimes called dorsal anterior cingulate) cortices were specific to BD who showed the smaller difficulty-related changes in these regions than individuals with UD. In addition, the changes in the lateral occipital cortex were significantly associated with the difficulty-related changes in RT in BD. The lateral occipital cortex is important for object recognition [75], visual imagery [76], and response to emotional vs. neutral visual stimuli [77]. Recently, the occipital cortex started receiving attention in the context of BD. It was shown that individuals with BD have increased asymmetry [78], increased grey matter volume [79], and reduced ability to longitudinally adjust activation in these regions during anticipation of emotionally negative events [80]. The paracingulate cortex is critical for monitoring cognitive interference [81] and efficient target identification in the n-back tasks [82]. Previous studies revealed that this region had reduced cortical thickness in individuals with BD [83] and was important for BD/UD classification in the n-back task [32]. These results, taken together with the findings that individuals with BD had significantly lower accuracy in the 2-back task than those with UD, suggest that aberrant activation in the left lateral occipital and right paracingulate cortices may be a reason for diminished behavioral performance in the former. Specifically, individuals with BD may have lower working memory capacity than those with UD.

There were several limitations to this study. First, the experimental paradigm required participants to respond to targets only (which was only 30% of trials) thus limiting our ability to interpret incorrect responses as well as the processing of non-target items. Second, although the data were collected in the same laboratory using the same task, participants were scanned using two different scanners. We attempted to resolve this issue by orthogonalizing the design matrix to remove variance in the stepwise manner. Finally, results derived from secondary data analysis are limited by potential cohort effects within individual datasets.

In summary, our study has demonstrated that individuals with BD, UD, and HC differed in activation of the working memory circuitry during working memory updating. Difficulty-related activation changes in the left lateral occipital cortex and right paracingulate cortex were lower in individuals with BD, compared to those with UD. The left lateral occipital cortex function diminished with age and was associated with difficulty-related worsening of RT in BD but not in the other groups. Our findings are consistent with the proposal that the alterations in the working memory circuitry may be a trait characteristic of reduced working memory capacity in mood disorders, especially in BD. Aberrant patterns of difficulty-related response in the left lateral occipital and right paracingulate cortices could point to a specific marker of bipolar disorder.

## Supplementary information

Supplemental materials

Supplemental Tables
