# Supplementary material for: Working memory updating in individuals with bipolar and unipolar depression: fMRI study
Source: Transl Psychiatry. 2022 Oct 11;12:441. doi: 10.1038/s41398-022-02211-6 (PMC9553934; doi:10.1038/s41398-022-02211-6)
Supplement: Supplementary file 1 — Supplemental materials [file 41398_2022_2211_MOESM1_ESM.docx]

# SUPPLEMENTAL MATERIALS

## SUPPLEMENTAL FIGURES


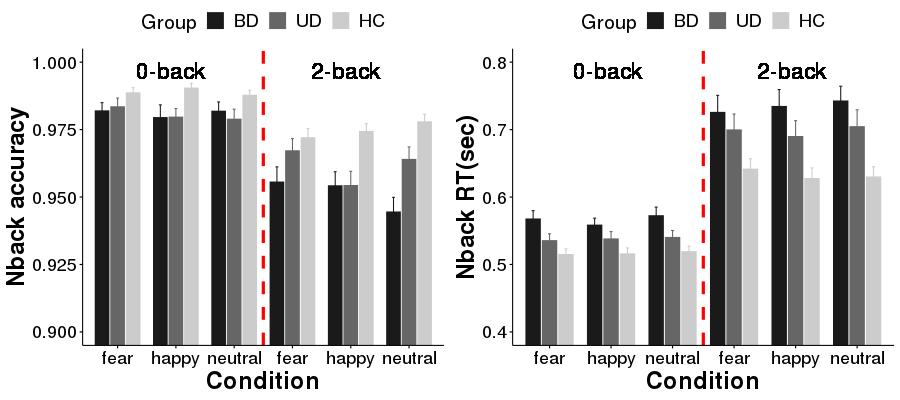


**Figure S1.** The n-back task accuracy and RT (accurate responses only) for individuals with BD, UD, and HC.


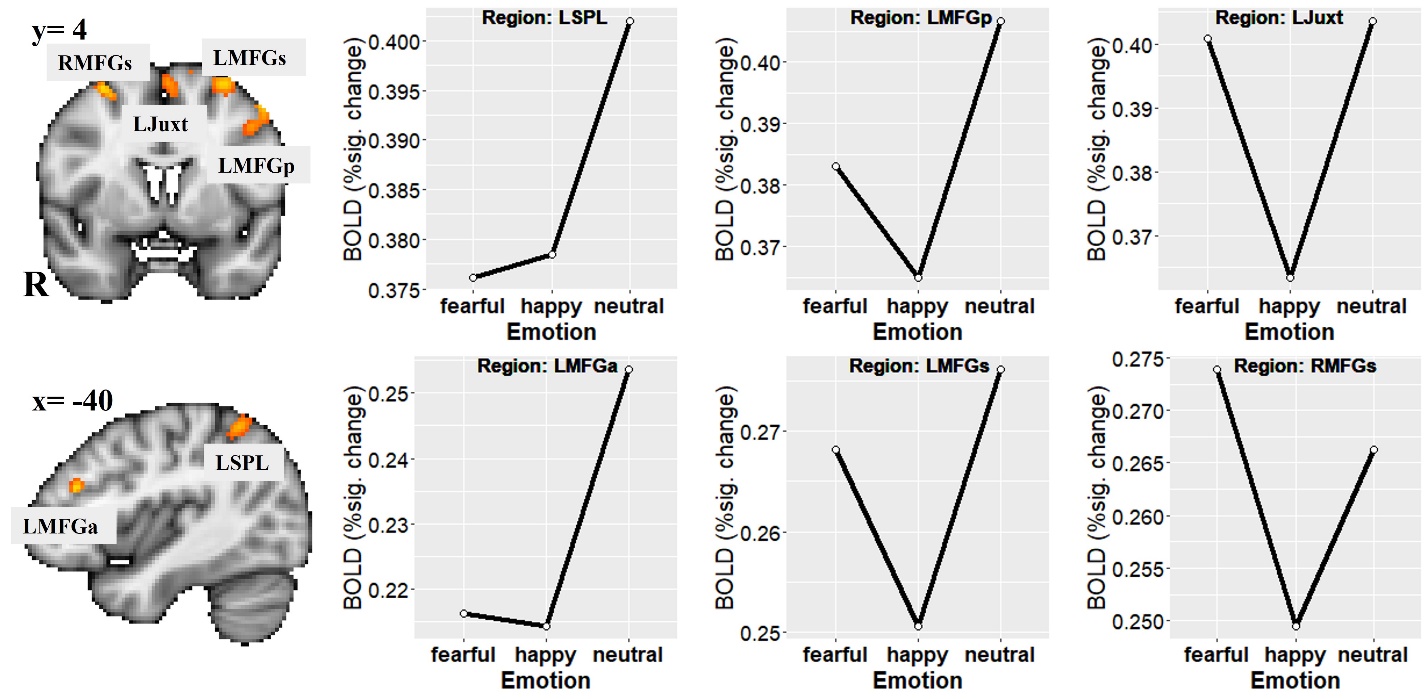


**Figure S2.** The main effect of emotions on the 2-back minus 0-back activation differences (voxel-wise corrected at p<0.01) in the left superior parietal lobule (LSPL), left middle frontal gyrus posterior (LMFGp), left juxtaposition lobule (LJuxt), left middle frontal gyrus anterior (LMFGa), and left and right left middle frontal gyrus superior (LMFGs and RMFGs).


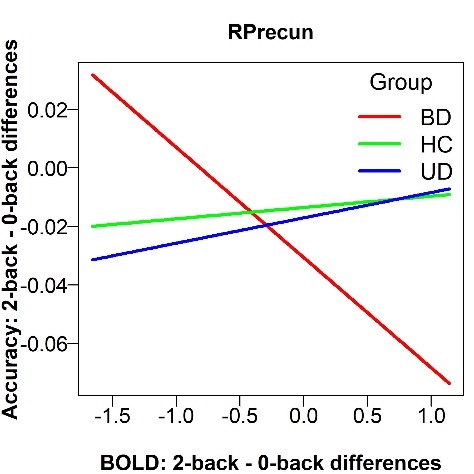


**Figure S3**. The relationship between the 2-back minus 0-back activation differences in the right precuneus (RPrecun) and the 2-back minus 0-back differences in accuracy.


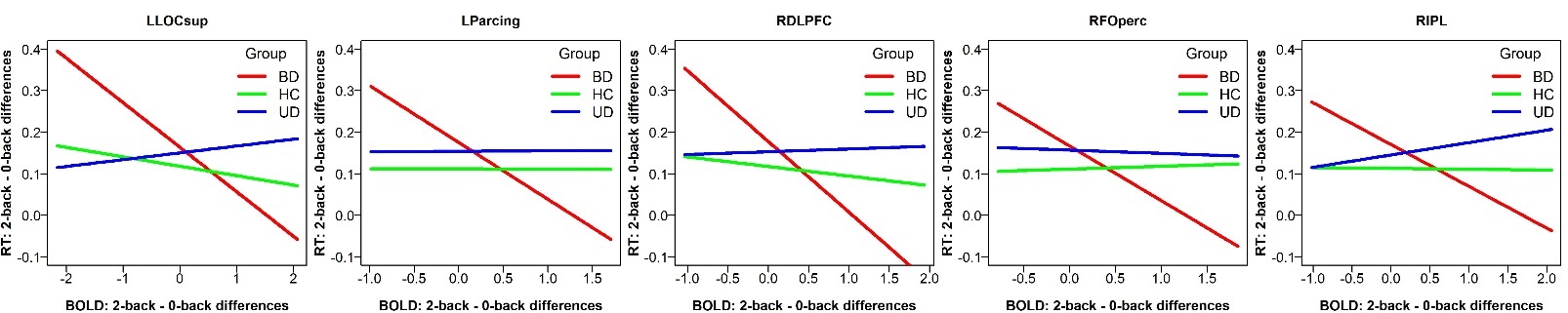


**Figure S4**. The relationship between the 2-back minus 0-back differences in RT and the 2-back minus 0-back activation differences in the left lateral occipital cortex superior division (LLOCsup), left paracingulate cortex (LParacing), right dorsolateral prefrontal cortex (RDLPFC), right frontal opercular cortex (RFOperc) and right inferior parietal lobule (RIPL).
